# Long-Term Real-World Effectiveness and Response Trajectories of Dupilumab in Paediatric Atopic Dermatitis

**DOI:** 10.3390/jcm15134862

**Published:** 2026-06-23

**Authors:** Małgorzata Ponikowska, Emilia Kucharczyk, Karol Biliński, Danuta Nowicka, Łukasz Lewandowski

**Affiliations:** 1 Centre of General Dermatology and Oncodermatology, Wroclaw Medical University, 50-556 Wroclaw, Poland; danuta.nowicka@umw.edu.pl; 2 University Centre of General and Oncological Dermatology, University Clinical Hospital in Wroclaw, Borowska 213, 50-556 Wroclaw, Poland; 3 Faculty of Medicine, Wroclaw Medical University, 50-367 Wroclaw, Poland; 4 Department of Biochemistry and Immunochemistry, Faculty of Medicine, Wroclaw Medical University, 50-368 Wroclaw, Poland; lukasz.lewandowski@umw.edu.pl

**Keywords:** atopic dermatitis, dupilumab, real-world evidence, EASI, biomarkers

## Abstract

**Background/Objectives:** Long-term real-world data on dupilumab effectiveness and response trajectories in paediatric atopic dermatitis (AD) remain limited. This study evaluated long-term effectiveness, safety, response durability, and treatment trajectories in paediatric patients with moderate-to-severe AD treated with dupilumab. **Methods:** This retrospective single-centre cohort study included 55 paediatric patients with moderate-to-severe AD treated with dupilumab. Clinical outcomes, including Eczema Area and Severity Index (EASI), body surface area (BSA), and Children’s Dermatology Life Quality Index (CDLQI), were assessed longitudinally throughout treatment. The primary endpoint was the achievement of EASI-75 over the course of treatment, including timing of response onset. Secondary endpoints included EASI-90 achievement, longitudinal changes in disease severity and quality of life, treatment durability, safety, and response trajectory analyses. Patients were additionally classified into mutually exclusive trajectory groups based on timing and durability of EASI-75 achievement. **Results:** Dupilumab treatment was associated with rapid and sustained clinical improvement. Some patients achieved EASI-75 as early as week 4, highlighting interindividual variability in response kinetics. At week 16, EASI-75 was achieved in 85.5% of patients and EASI-90 in 47.3%. Clinical effectiveness further improved during long-term follow-up, with EASI-75 achieved in 96.2% and EASI-90 in 86.5% of patients at the last available follow-up. Median CDLQI decreased from 22.0 at baseline to 3.0 during follow-up, indicating marked improvement in quality of life. Most patients (83.6%) were classified as early responders, while 10.9% demonstrated delayed but clinically meaningful improvement during continued treatment. Loss of response after initial EASI-75 achievement was uncommon (3.7%). Adverse events were predominantly mild, with eosinophilia and conjunctivitis representing the most frequently observed findings. **Conclusions:** Overall, dupilumab demonstrated high long-term effectiveness and favourable tolerability in paediatric patients with moderate-to-severe AD. Trajectory analyses revealed clinically meaningful heterogeneity in response kinetics despite favourable long-term outcomes, highlighting that delayed responders may still achieve substantial benefit during continued therapy.

## 1. Introduction

Atopic dermatitis (AD) is one of the most common chronic inflammatory skin diseases in children and adolescents, with increasing global prevalence over recent decades [1]. Moderate-to-severe paediatric AD is associated with substantial disease burden, including intense pruritus, sleep disturbance, impaired psychosocial functioning, and reduced quality of life affecting both patients and their families [2]. In addition to its cutaneous manifestations, persistent uncontrolled inflammation during childhood may negatively influence emotional well-being, social functioning, and educational performance [3].

Although topical therapies remain the foundation of AD management, a substantial proportion of paediatric patients require systemic treatment because of inadequate disease control [4]. Dupilumab, a fully human monoclonal antibody targeting the IL-4 receptor α subunit and inhibiting IL-4/IL-13 signalling, has fundamentally changed the therapeutic landscape of moderate-to-severe AD [5]. Owing to its favourable efficacy and safety profile, dupilumab has become the first-line biologic therapy in paediatric AD and currently remains the only biologic approved for children as young as 6 months of age [6,7,8].

Randomised clinical trials have demonstrated significant clinical improvement and acceptable safety of dupilumab across paediatric age groups [8]. However, real-world data evaluating long-term treatment effectiveness, durability of response, and clinically relevant response trajectories in children remain comparatively limited, particularly outside controlled trial settings. Furthermore, predictors of treatment response in paediatric AD have not been clearly established, and the clinical relevance of routinely available inflammatory biomarkers remains uncertain.

Given the growing use of dupilumab in paediatric dermatology, better characterisation of long-term treatment patterns and response heterogeneity may have important implications for clinical management and individualised monitoring strategies. Therefore, the aim of this study was to evaluate the long-term real-world effectiveness and safety of dupilumab in children and adolescents with moderate-to-severe AD, with particular emphasis on response trajectories, durability of response, and exploratory predictors of clinical outcomes.

## 2. Materials and Methods

### 2.1. Study Design

This was a single-centre, retrospective cohort study conducted at University Centre Wroclaw Medical University. The study included paediatric patients with moderate-to-severe atopic dermatitis (AD) who initiated dupilumab treatment between the ages of 3 and 17 years. Clinical data were collected from medical records at baseline (week 0) and during follow-up visits at weeks 16, 32, and 52, as well as at the last available follow-up visit.

### 2.2. Participants

#### 2.2.1. Inclusion Criteria

Eligible participants were children and adolescents aged ≥6 months and <18 years with a confirmed diagnosis of moderate-to-severe AD who initiated dupilumab treatment during the study period. Follow-up duration varied across the cohort. In patients who discontinued treatment or were lost to further follow-up, data from the last available clinical visit were included in the final analysis.

#### 2.2.2. Exclusion Criteria

Patients were excluded if they: received systemic immunosuppressive or corticosteroid therapy during dupilumab treatment, had incomplete medical data or missed follow-up visits, or had concomitant chronic dermatologic or systemic inflammatory diseases that could affect AD assessment.

### 2.3. Data Collection and Baseline Characteristics

Baseline data included demographic characteristics, disease duration, clinical severity measures (EASI, CDLQI, and BSA), atopic comorbidities, previous systemic treatment exposure, and laboratory parameters. Exploratory biomarkers included absolute eosinophil count, total serum IgE, C-reactive protein (CRP), and eosinophil-to-lymphocyte ratio (ELR). Adverse events recorded during treatment were also collected from medical records.

### 2.4. Treatment Protocol

Dupilumab dosing followed approved age- and weight-based regimens according to current prescribing recommendations [9].

### 2.5. Outcomes

Clinical outcomes were assessed at weeks 16, 32, 52, and the last available follow-up. Treatment effectiveness was evaluated using percentage reduction in EASI score from baseline and standard response thresholds (EASI-50, EASI-75, EASI-90, and EASI-100). Additional outcomes included changes in BSA and CDLQI.

Patients were classified into mutually exclusive trajectory groups based on timing and durability of EASI-75 achievement. Early responders were defined as patients achieving EASI-75 within the first 16 weeks of treatment. Late responders achieved EASI-75 after week 16 during continued therapy. Non-responders did not achieve EASI-75 at any evaluated timepoint. Loss of response was defined as initial achievement of EASI-75 followed by subsequent loss of this response during follow-up.

Safety outcomes were assessed descriptively using data extracted from medical records.

### 2.6. Statistical Analysis

All analyses were performed in R (R Foundation for Statistical Computing, Vienna, Austria) following a predefined analytical workflow including data preprocessing, descriptive clinical analyses, and predictive modelling.

#### 2.6.1. Variable Definition and Preprocessing

The updated dataset, including extended follow-up to week 52, was analysed as a single longitudinal cohort. Age at atopic dermatitis (AD) onset and time from disease onset to treatment initiation were derived from source records. The eosinophil-to-lymphocyte ratio (ELR) was calculated from absolute eosinophil and lymphocyte counts.

A complementary classification based on maximum depth of response achieved during follow-up was additionally used for descriptive analyses.

#### 2.6.2. Descriptive Analyses

Baseline characteristics were summarised using medians with interquartile ranges (IQRs) for continuous variables and counts with percentages for categorical variables. Clinical outcomes were summarised at week 16, week 32, week 52, and the last follow-up. These included EASI, percentage EASI reduction, CDLQI, BSA, and proportions of patients achieving EASI response thresholds. Durability of response was assessed descriptively using trajectory-based classifications and proportions of patients maintaining EASI-75 across follow-up. Depth of response was summarised using maximum achieved response categories. Safety outcomes were summarised descriptively as the proportion of patients with any adverse event and frequencies of reported event types.

#### 2.6.3. Predictive Modelling

Exploratory regression analyses were performed to evaluate potential baseline correlates of treatment response, including EASI-75 and EASI-90 at week 16, early response status, and achievement of EASI-100 during follow-up. Logistic regression models were adjusted for clinically relevant baseline variables.

#### 2.6.4. Missing Data

No imputation was performed. Analyses were conducted using complete-case datasets defined separately for each model. Consequently, sample size varied across analyses depending on data availability.

## 3. Results

### 3.1. Descriptive Statistics

#### 3.1.1. Cohort Characteristics

The study cohort comprised 55 paediatric patients with moderate-to-severe atopic dermatitis treated with dupilumab. Most patients were followed for longer than one year, with clinical response data available for 55 patients at week 16, 53 at week 32, 51 at week 52, and 52 at the last available follow-up. Baseline demographic and clinical characteristics are summarised in Table 1. The median follow-up duration was 102.5 weeks (IQR 88–128).

The median age at treatment initiation was 11.0 years (IQR 8.5–12.5), while the median age at AD onset was 1.0 year (IQR 0.25–3.00), reflecting predominantly early-onset disease. The median time from disease onset to initiation of dupilumab treatment was 9.0 years (IQR 6.15–10.75), indicating a cohort with long-standing and persistent disease burden.

At baseline, patients demonstrated severe disease activity, with a median EASI of 22.0 (IQR 20.6–27.6), CDLQI of 22.0 (IQR 18.0–25.0), and BSA involvement of 28.0% (IQR 19.0–44.5). Elevated type 2 inflammatory markers were commonly observed, including a median eosinophil count of 0.64 × 10^3^/L (IQR 0.51–1.11) and median total IgE concentration of 1452 IU/mL (IQR 331.5–5175.0).

Atopic comorbidities were frequent, particularly airborne allergy (76.4%) and food allergy (60.0%). Most patients had previously received systemic therapy, including cyclosporine in 58.2% of cases, whereas prior JAK inhibitor exposure was uncommon.

#### 3.1.2. Effectiveness over Time

Dupilumab treatment resulted in rapid and sustained clinical improvement across all evaluated outcome measures. By week 16 (N = 55), median EASI decreased from 22.0 at baseline to 2.4 (IQR 0.9–4.9), corresponding to a median reduction of 89.1% (IQR 81.5–95.2). Clinically meaningful response was achieved in most patients, with EASI-75 observed in 85.5% and EASI-90 in 47.3% of the cohort. All patients achieved at least EASI-50 at this timepoint.

Clinical effectiveness continued to improve throughout follow-up. Median EASI further decreased to 1.9 (IQR 1.1–3.4) at week 32 and 1.6 (IQR 0.2–2.9) at week 52. At the last available follow-up (N = 52), disease control remained consistently high, with a median EASI of 0.9 (IQR 0.2–2.0) and a median overall EASI reduction of 96.6% (IQR 93.3–99.0). At this final timepoint, EASI-75 was achieved in 96.2% of patients, EASI-90 in 86.5%, and complete skin clearance (EASI-100) in 21.2%.

Improvements in physician-assessed disease severity were paralleled by marked reductions in disease extent and quality-of-life impairment. Median CDLQI decreased from 22.0 at baseline to 6.0 at week 16 and further improved to 3.0 at the last available follow-up. Similarly, median BSA decreased from 28.0% at baseline to 3.0% at week 16 and remained low during long-term observation.

Overall, treatment responses were highly durable. During follow-up, 54/55 patients (98.2%) achieved EASI-75 at least once, with most responses occurring early after treatment initiation.

#### 3.1.3. Response Trajectories

Patients were classified into clinically defined response trajectories based on the timing and durability of EASI-75 achievement (Table 2). Most patients were classified as early responders, with 46/55 patients (83.6%) achieving EASI-75 by week 16 without subsequent loss of response. A smaller subgroup of six patients (10.9%) demonstrated delayed but clinically meaningful improvement and were classified as late responders. Loss of response after prior EASI-75 achievement occurred in only two patients (3.6%), while one patient (1.8%) did not achieve EASI-75 during follow-up.

Early responders demonstrated rapid and sustained disease control throughout follow-up, with progressive deepening of response over time. In contrast, late responders showed slower but continued clinical improvement, with all patients achieving EASI-75 by week 52. Deep responses, including EASI-90 and EASI-100, also became increasingly frequent during prolonged treatment exposure.

Patients with loss of response initially demonstrated improvement comparable to early responders, followed by transient deterioration in disease control during later follow-up. The single non-responder demonstrated partial but persistently insufficient clinical improvement throughout observation.

Overall, these findings indicate clinically relevant heterogeneity of treatment trajectories despite uniformly high overall effectiveness.

#### 3.1.4. Depth of Response

Deep clinical responses were common during long-term dupilumab treatment. EASI-90 represented the most frequently achieved maximum response, while complete skin clearance (EASI-100) was observed in 34.5% of patients. Only one patient did not achieve EASI-75 during follow-up, and loss of response remained uncommon.

#### 3.1.5. Safety

Adverse events were reported in 16/54 patients (29.6%) and were predominantly mild in severity. The most frequently observed finding was eosinophilia, reported in nine patients, including cases accompanied by additional laboratory abnormalities. Conjunctivitis occurred in three patients, while upper respiratory tract infections and neutropenia-related findings were each observed in two patients. Other adverse events were isolated.

No consistent pattern of severe or clinically limiting toxicity was identified during follow-up. Treatment discontinuation or switching occurred in six patients (10.9%). Reasons included primary non-response (*n* = 1), secondary loss of response (*n* = 2), treatment-related eyelid oedema (*n* = 1), and loss to follow-up following relocation abroad (*n* = 2). Detailed reasons for treatment discontinuation or switching are presented in Supplementary Table S1. Laboratory-related abnormalities requiring clinical attention were documented in three patients (5.5%).

Overall, the safety profile observed in this cohort was consistent with the known tolerability profile of dupilumab in paediatric AD. A summary of adverse events observed during treatment is presented in Table 3.

### 3.2. Exploratory Predictors of Response

Exploratory predictive analyses suggested that greater baseline inflammatory burden may be associated with slower early response kinetics (Table 4). Higher baseline disease severity demonstrated the most consistent inverse association with achievement of EASI-75 and EASI-90 at week 16. Similarly, higher eosinophil-related markers, including eosinophil-to-lymphocyte ratio (ELR) and absolute eosinophil count, showed inverse trends in relation to early treatment response. Higher total IgE levels also demonstrated directionally similar associations in selected models. However, effect estimates remained modest and imprecise.

Moderate atopic burden was additionally associated with lower likelihood of achieving EASI-100 during follow-up, although the absence of a clear gradient across burden categories limits interpretation of this finding. Overall, no baseline variable demonstrated sufficiently robust predictive performance to support clinically applicable treatment stratification, and all predictive analyses should therefore be regarded as exploratory and hypothesis-generating.

## 4. Discussion

In this real-world paediatric cohort, dupilumab demonstrated substantial and sustained effectiveness across multiple clinically relevant domains, including physician-assessed disease severity, body surface area involvement, and quality of life. Most patients achieved rapid clinical improvement, with EASI-75 observed in 85.5% at week 16 and maintained or further improved during long-term follow-up. Treatment responses were rapid and durable, with most patients eventually achieving EASI-75 and many reaching EASI-90 or EASI-100. These findings support the growing body of evidence indicating that dupilumab represents an effective long-term therapeutic option for paediatric patients with moderate-to-severe AD in routine clinical practice.

The overall effectiveness observed in our cohort appears comparable or even numerically higher than that reported in pivotal clinical trials and previously published real-world studies [10,11,12]. In the LIBERTY AD clinical program, EASI-75 responses at week 16 ranged from approximately 53% to 70%, depending on age group and dosing regimen [12,13]. The higher response rates observed in our cohort may partly reflect differences between controlled trial populations and routine clinical practice, including longer observation and individualised treatment approaches. Importantly, continued improvement during follow-up suggests that some paediatric patients may achieve progressively deeper responses beyond the conventional short-term assessment window.

Given the broad age range of our cohort, we additionally explored treatment outcomes according to age at dupilumab initiation. Although adolescents had a later age at the disease onset and a longer interval between AD onset and initiation of biologic therapy, treatment effectiveness was remarkably similar across age groups. No significant differences were observed in EASI-75, EASI-90, or early response rates at week 16, and long-term disease control remained high in both children and adolescents. These findings suggest that dupilumab maintains robust effectiveness across paediatric age groups despite potential age-related differences in disease phenotype and immune activation patterns.

An additional clinically relevant observation was the marked heterogeneity of treatment trajectories despite uniformly high overall effectiveness. While most patients achieved rapid improvement, a smaller subgroup demonstrated delayed response or fluctuating disease control, supporting biological heterogeneity of paediatric AD [13,14]. Recognition of distinct response patterns may have implications for patient counselling and long-term monitoring strategies. In this context, trajectory-based analyses may provide clinically meaningful information beyond conventional single-timepoint endpoints.

Importantly, many patients classified as late responders ultimately achieved disease control comparable to that observed in early responders. This finding may have practical clinical implications, suggesting that lack of rapid improvement should not necessarily prompt premature discontinuation of dupilumab, particularly in the absence of disease worsening or significant safety concerns [15]. At the same time, the identification of a small subgroup with loss of response highlights that long-term disease control may fluctuate even during continued IL-4/IL-13 blockade. Such variability likely reflects the biological complexity of AD and interindividual differences in inflammatory endotypes, barrier dysfunction, environmental exposures, and treatment adherence.

The observed clinical improvement was accompanied by a profound reduction in disease-related quality-of-life impairment, with CDLQI scores improving rapidly after treatment initiation and remaining low throughout follow-up. Given the substantial psychosocial burden of paediatric AD, including sleep disturbance, emotional distress, impaired social functioning, and negative effects on family quality of life, these findings highlight that the benefits of dupilumab extend beyond skin clearance alone [2]. The durability of both clinical and quality-of-life improvement observed in our cohort is particularly relevant in the paediatric population, in whom chronic uncontrolled disease may interfere with psychosocial development, educational functioning, and family dynamics.

Exploratory predictive analyses suggested that greater baseline inflammatory burden may be associated with slower early response kinetics. Higher baseline EASI consistently demonstrated inverse associations with achievement of EASI-75 and EASI-90 at week 16, although long-term outcomes remained highly favourable even among patients with initially severe disease. Similarly, higher eosinophil-related inflammatory markers, including absolute eosinophil count and ELR, were associated with reduced likelihood of achieving early clinical response [16,17]. These findings should not be interpreted as evidence of true biological resistance to dupilumab. Rather, eosinophilia in AD may reflect a broader and more immunologically complex type 2 inflammatory phenotype associated with slower response kinetics rather than reduced ultimate responsiveness to treatment [14]. This interpretation appears particularly relevant in our cohort, where most patients ultimately achieved sustained high-level responses despite variability in early treatment dynamics. Similar observations have been reported inconsistently in previous real-world studies, highlighting the ongoing uncertainty regarding the predictive role of eosinophil-related biomarkers in AD [14]. However, the precision of these associations remained limited, and these findings should therefore be regarded as exploratory and hypothesis-generating rather than clinically applicable predictors at present. However, these findings should be considered exploratory and hypothesis-generating rather than clinically applicable predictors at present.

Total IgE also demonstrated inverse trends in relation to early response and achievement of EASI-100. Elevated IgE is a well-recognised feature of severe type 2 inflammation in AD, although its utility as a predictive biomarker remains uncertain [16]. Some previous studies have suggested that very high IgE levels may correlate with delayed improvement or greater disease chronicity, whereas others have found limited predictive value. Our findings contribute to this discussion but do not support the use of baseline IgE alone as a clinically meaningful predictor of dupilumab response.

Interestingly, moderate atopic burden was associated with lower odds of achieving EASI-100. However, the absence of a clear dose–response relationship across burden categories limits interpretation of this finding. It is possible that coexistence of multiple atopic comorbidities reflects broader immune dysregulation, potentially influencing the likelihood of complete skin clearance even under targeted cytokine inhibition. Nevertheless, given the limited sample size and exploratory nature of these analyses, this observation requires confirmation in larger cohorts.

An exploratory subgroup analysis according to asthma status revealed that patients with concomitant asthma had higher baseline eosinophil counts and numerically less favourable treatment outcomes across several response measures. In particular, lower EASI-75 and EASI-90 response rates were observed at selected timepoints compared with patients without asthma. Although these findings should be interpreted cautiously because of the small number of patients with asthma and the exploratory nature of the analysis, they may suggest that a broader type 2 inflammatory burden is associated with slower treatment kinetics. Further studies are needed to determine whether asthma or other atopic comorbidities independently influence dupilumab responsiveness in paediatric AD.

The safety profile observed in our cohort was consistent with previous paediatric and adult studies of dupilumab. Most adverse events were mild and manageable, with eosinophilia and conjunctivitis representing the most frequently observed findings. Importantly, no consistent signal of severe systemic toxicity emerged during extended follow-up. Treatment discontinuation was uncommon, further supporting the favourable long-term tolerability of dupilumab in paediatric clinical practice.

This study has several strengths. First, it provides long-term real-world data in a paediatric population spanning a broad age range, including younger children who remain underrepresented in many observational studies. Second, the trajectory-based analytical approach allowed characterisation of clinically meaningful response patterns beyond conventional single-timepoint outcomes. Third, the study incorporated both clinician-assessed and patient-reported outcomes, allowing comprehensive evaluation of long-term treatment benefit.

Several limitations should also be acknowledged. The retrospective single-centre design introduced risk of selection bias and incomplete data capture. The limited sample size reduced statistical power for predictive analyses, particularly for uncommon outcomes such as non-response or loss of response. In addition, response timing was based on visit-level assessments and adverse-event reporting relied on routine clinical documentation. Additionally, subgroup analyses according to age and asthma status were exploratory in nature and involved relatively small patient numbers, particularly in the asthma subgroup. Therefore, these findings should be considered hypothesis-generating and require confirmation in larger multicentre cohorts.

Despite these limitations, our findings reinforce the role of dupilumab as an effective and durable treatment for paediatric AD in routine clinical practice. The consistently high long-term response rates observed in this cohort support early consideration of targeted biologic therapy in appropriately selected children with moderate-to-severe disease. Future multicentre studies integrating longitudinal clinical trajectories with biomarker profiling may help better define predictors of treatment kinetics, durability of response, and mechanisms underlying delayed response or secondary loss of efficacy.

## 5. Conclusions

In this real-world paediatric cohort, dupilumab demonstrated high long-term effectiveness and favourable tolerability in children and adolescents with moderate-to-severe atopic dermatitis. Most patients achieved rapid and durable disease control, accompanied by sustained improvement in quality of life. Although most responses occurred early after treatment initiation, a smaller subgroup demonstrated delayed but clinically meaningful improvement during continued therapy. These findings highlight clinically relevant heterogeneity of treatment trajectories and suggest that lack of rapid early response should be interpreted cautiously in selected patients. Further multicentre longitudinal studies are needed to better define predictors of treatment kinetics, durability of response, and long-term disease control in paediatric AD.

## Figures and Tables

**Table 1:** Baseline characteristics of the study cohort.

Variable	*n*	Overall Cohort
Demographics		
Age at program entry, years	55	11.0 [8.5, 12.5]
Weight, kg	55	37.5 [30.0, 46.2]
Weight centile	55	50.0 [23.5, 85.0]
Height, cm	55	140.0 [131.0, 155.0]
Height centile	55	43.0 [15.0, 75.5]
Disease history		
Age at onset of AD, years	55	1.00 [0.25, 3.00]
Time from onset to treatment, years	54	9.00 [6.15, 10.75]
Atopic comorbidities		
Asthma	54	10 (18.5%)
Allergic rhinitis	55	7 (12.7%)
Food allergy	55	33 (60.0%)
Airborne allergy	55	42 (76.4%)
Any drug allergy	55	5 (9.1%)
Atopic burden		
Atopic burden, *n*	55	2.0 [1.0, 2.0]
Low	55	18 (32.7%)
Moderate	55	26 (47.3%)
High	55	11 (20.0%)
Treatment		
Prior cyclosporine	55	32 (58.2%)
Prior JAK inhibitor	55	2 (3.6%)
Dupilumab dose at baseline, mg	55	300 [300, 400]
Disease severity		
EASI	55	22.0 [20.6, 27.6]
CDLQI	55	22.0 [18.0, 25.0]
BSA, %	55	28.0 [19.0, 44.5]
Laboratory markers Eosinophils, absolute (×10^3^/L)	54	0.64 [0.51, 1.11]
Eosinophils, %	54	9.2 [6.6, 12.4]
Lymphocytes, absolute (×10^3^/L)	52	2.42 [1.89, 3.05]
WBC (×10^3^/L)	55	7.67 [6.40, 9.16]
Total IgE, IU/mL	55	1452.0 [331.5, 5175.0]
CRP, mg/L	55	0.50 [0.00, 1.30]
ELR	52	0.272 [0.182, 0.448]
Safety		
Any adverse event	54	16 (29.6%)

Data are presented as median [interquartile range] for continuous variables and *n* (%) for categorical variables. Percentages were calculated using the number of non-missing observations shown in the “*n*” column. Abbreviations: AD—atopic dermatitis; EASI—Eczema Area and Severity Index; CDLQI—Children’s Dermatology Life Quality Index; BSA—body surface area; WBC—white blood cell count; ELR—eosinophil-to-lymphocyte ratio.

**Table 2:** Longitudinal outcomes by response trajectory.

Trajectory	Time *n*		EASI	∆EASI (%)	CDLQI	EASI-75	EASI-90	EASI-100
Early (*n* = 46, 83.6%)	w16	46	2.0 [0.8, 3.6]	92.2 [84.3, 96.2]	5.5 [2.0, 8.0]	97.8%	54.3%	15.2%
	w32	44	1.5 [0.5, 2.7]	93.5 [88.6, 97.7]	4.0 [2.0, 7.0]	95.5%	68.2%	13.6%
	w52	42	1.3 [0.1, 2.3]	94.5 [90.4, 99.7]	3.0 [2.0, 6.0]	95.2%	78.6%	21.4%
	last	43	0.7 [0.2, 1.3]	97.5 [94.4, 99.0]	2.0 [1.0, 5.0]	100%	90.7%	20.9%
Late (*n* = 6, 10.9%)	w16	6	10.3 [9.5, 12.2]	66.8 [59.4, 68.5]	9.5 [8.2, 10.8]	0%	0%	0%
	w32	6	6.6 [4.3, 7.4]	78.3 [72.7, 86.2]	8.0 [6.5, 9.5]	50.0%	16.7%	0%
	w52	6	3.0 [2.1, 3.0]	90.1 [88.4, 93.2]	6.0 [5.2, 6.0]	100%	50.0%	0%
	last	6	2.0 [0.5, 2.0]	94.5 [91.6, 99.1]	4.0 [1.0, 6.2]	83.3%	83.3%	33.3%
Loss of response (*n* = 2, 3.6%)	w16	2	3.8 [3.1, 4.6]	85.4 [82.8, 87.9]	5.0 [5.0, 5.0]	100%	50.0%	0%
	w32	2	2.8 [2.8, 2.8]	89.2 [89.1, 89.4]	2.0 [1.5, 2.5]	100%	0%	0%
	w52	2	8.8 [5.6, 11.9]	65.6 [53.0, 78.1]	10.0 [9.5, 10.5]	50.0%	50.0%	0%
	last	2	3.4 [2.3, 4.5]	86.7 [82.2, 91.1]	5.5 [3.2, 7.8]	100%	50.0%	0%
Non-response (*n* = 1, 1.8%)	w16	1	11.1 [11.1, 11.1]	59.0 [59.0, 59.0]	5.0 [5.0, 5.0]	0%	0%	0%
	w32	1	20.4 [20.4, 20.4]	24.7 [24.7, 24.7]	19.0 [19.0, 19.0]	0%	0%	0%
	w52	1	10.0 [10.0, 10.0]	63.1 [63.1, 63.1]	11.0 [11.0, 11.0]	0%	0%	0%
	last	1	20.4 [20.4, 20.4]	24.7 [24.7, 24.7]	19.0 [19.0, 19.0]	0%	0%	0%

Values are median [IQR] or percentages. EASI-75, EASI-90, and EASI-100 denote a ≥75%, ≥90%, and 100% reduction in EASI score from baseline. Abbreviations: EASI—Eczema Area and Severity Index; CDLQI—Children’s Dermatology Life Quality Index; EASI-75—≥75% reduction in EASI score from baseline; EASI-90—≥90% reduction in EASI score from baseline; EASI-100—100% reduction in EASI score from baseline.

**Table 3:** Summary of adverse events.

Adverse Event Category	N
No adverse event	38
Eosinophilia (including mixed laboratory findings)	9
Conjunctivitis	3
Upper respiratory tract infection	2
Neutropenia	2
Other (single events)	2

Categories were derived from raw clinical notes and grouped for descriptive purposes. Individual events may overlap across categories. Counts reflect the number of patients with a given event category, and categories are not mutually exclusive.

**Table 4:** Selected multivariable models for clinically oriented outcomes.

Outcome/Model	Predictor	Effect Estimate	*p*-Value	Sign Consistency
Early responder vs. non-early, core	Baseline EASI	0.949 (0.890 to 1.008)	0.088	97.6%
Early responder vs. non-early, core	Age at AD onset	0.896 (0.675 to 1.227)	0.446	93.6%
Early responder vs. non-early, core + IgE	Log_2_ total IgE	0.780 (0.476 to 1.092)	0.165	99.6%
Early responder vs. non-early, core + ELR	ELR	0.446 (0.094 to 2.473)	0.309	76.0%
Ever EASI-100 vs. not, core	Male sex	0.365 (0.104 to 1.197)	0.097	95.2%
Ever EASI-100 vs. not, core + atopic burden	Atopic burden: moderate vs. low	0.203 (0.046 to 0.765)	0.018	99.6%
Ever EASI-100 vs. not, core + atopic burden	Atopic burden: high vs. low	0.468 (0.082 to 2.403)	0.363	85.6%
Ever EASI-100 vs. not, core + IgE	Log_2_ total IgE	0.821 (0.639 to 1.019)	0.074	96.0%

Effect estimates are presented as odds ratios (ORs) with 95% confidence intervals. Sign consistency denotes the proportion of bootstrap resamples with the same coefficient direction as in the original model. The core model included age at AD onset, sex, baseline EASI, and time from onset to treatment initiation. Abbreviations: ELR—eosinophil-to-lymphocyte ratio; IgE—immunoglobulin E; EASI—Eczema Area and Severity Index; AD—atopic dermatitis.

## Data Availability

The data that support the findings of this study are available from the corresponding author upon reasonable request.

## Supplementary Materials

The following supporting information can be downloaded at: https://www.mdpi.com/article/10.3390/jcm15134862/s1. Table S1: Detailed reasons for treatment discontinuation or switching.
