# Supplementary material for: Long-Term Real-World Effectiveness and Response Trajectories of Dupilumab in Paediatric Atopic Dermatitis
Source: J Clin Med. 2026 Jun 23;15(13):4862. doi: 10.3390/jcm15134862 (PMC13361447; doi:10.3390/jcm15134862)
Supplement: Supplementary file 1 [file jcm-15-04862-s001.zip › jcm-4371023-supplementary.pdf]

Supplementary Table S1. Detailed reasons for treatment discontinuation or switching

Patient	Reason for discontinuation/switching	AE-related	Outcome
1	Primary non response	No	Switched to alternative systemic therapy
2	Secondary loss of response	No	Switched to alternative systemic therapy
3	Secondary loss of response	No	Switched to alternative systemic therapy
4	Severe eyelid oedema	Yes	Switched to alternative systemic therapy
5	Lost to follow-up following relocation abroad	No	Follow-up unavailable
6	Lost to follow-up following relocation abroad	No	Follow-up unavailable
